# The impact on time between injury and semi-acute surgery for hand fractures after virtual fracture clinic implementation

**DOI:** 10.1177/17531934241268976

**Published:** 2024-08-21

**Authors:** Dorien A. Salentijn, Gijs J.A. Willinge, Marcel G.W. Dijkgraaf, Ruben N. van Veen

**Affiliations:** 1 Amsterdam Public Health, Amsterdam, The Netherlands; 2 Department of Trauma Surgery, OLVG, Amsterdam, The Netherlands; 3 Department of Epidemiology and Data Science, Amsterdam UMC – University of Amsterdam, Amsterdam, The Netherlands

**Keywords:** Hand fractures, metacarpal fractures, phalangeal fractures, operative treatment, virtual fracture clinic

## Abstract

The aim of this before-and-after study was to evaluate the implementation of a virtual fracture clinic (VFC) on the time between injury and surgery in patients presenting with a phalangeal or metacarpal fracture and in need of semi-acute surgical treatment. Between 1 January and 30 September 2018 (pre-VFC) and in the same period in 2022 (VFC), 101 and 113 patients were included, respectively. Before VCF implementation, the time between injury and surgery was 8.9 days (95% confidence interval [CI]: 8.1 to 9.6), while after VCF implementation it was 7.6 days (95% CI: 7.0 to 8.3). In 2018, 7% of operations were unacceptably delayed beyond 14 days from injury, which was reduced to 5% in 2022, despite patient-presentation delays of up to 10 days. VFC implementation was associated with a reduction in time until semi-acute surgery for phalangeal or metacarpal fractures and improved the quality of semi-acute surgery planning.

**Level of evidence:** Level III

## Introduction

Hand injuries are among the most common traumas seen in emergency departments (ED) in the Netherlands (Stam, 2022). Early intervention plays a major role in achieving optimal outcomes (Bosscha and Snellen, 1993; Meals and Meals, 2013; Peyronson et al., 2023). Ideally, hand injuries should be operated within a few days up to a week, with the possibility of extending the timeframe up to 10 days (Christodoulou et al., 2023; Yang et al., 2023). Operative treatment beyond 14 days seems undesirable as a result of ongoing fracture healing and prolonged immobilization (Bosscha and Snellen, 1993; Nellans and Chung, 2013; Tomlinson, 2012).

In the past few years, the healthcare system has faced mounting pressure due to population growth and an increase in the incidence of injuries. These challenges have been exacerbated by the COVID-19 pandemic, which placed an additional burden on healthcare resources and medical professionals (Beerekamp et al., 2017; Geerdink et al., 2020). Simultaneously, there has been a notable transition from non-operative approaches to operative treatment for hand injuries (Beerekamp et al., 2017; Edmonds et al., 2022; Merckaert et al., 2021; Ootes et al., 2012). In response, trauma care professionals developed the virtual fracture clinic (VFC).

The main purpose of a VFC is to enable efficient planning of follow-up treatment for patients with injuries (Geerdink et al., 2020; McKirdy and Imbuldeniya, 2017). Patients with injuries that require follow-up treatment, non-operative and operative, are referred to a VFC review. In this daily multidisciplinary meeting, the follow-up treatment, including the optimal planning of surgical intervention, is carefully designed under the direct supervision of an experienced orthopaedic trauma surgeon (Geerdink et al., 2021b).

Several studies have demonstrated positive outcomes regarding the effectiveness of planning and treatment in extremity-trauma-related care (i.e. ankle, foot, hand, wrist, elbow, knee) through this innovative VFC concept (Bhattacharyya et al., 2017; Cavka et al., 2021; Davey et al., 2020; Geerdink et al., 2021a, 2021b; Little et al., 2020; McKirdy and Imbuldeniya, 2017; Willinge et al., 2024). The aim of this study was to assess the impact on the time (in days) from injury to surgery in patients with phalangeal or metacarpal fractures requiring semi-acute operative treatment after the implementation of a VFC.

## Methods

### Design and setting

This retrospective cohort study was conducted at an urban level 2 trauma centre and teaching hospital in the Netherlands comprising two separate sites. Ethical approval was obtained by the institutional review board of our hospital (WO. 22. 143). The study received a waiver for informed consent due to its retrospective nature.

All consecutive patients were included if they: (1) presented to the hospital between 1 January and 30 September 2018 (pre-VFC implementation) or between 1 January and 30 September 2022 (during VFC implementation); (2) had a metacarpal or phalangeal fracture; (3) underwent semi-acute operative treatment. These time periods were selected to minimize bias related to the COVID-19 pandemic or seasonal influences and to skip any original adjustment period after the implementation of the new workflow in 2020. Patients were excluded if they: (1) required immediate surgical intervention (e.g. open fractures, nerve injuries and additional soft tissue injuries, such as tendon ruptures or severe skin lacerations); (2) experienced a surgical delay between the first hospital presentation and surgery of more than 14 days, unless this surgical delay was attributed to bad planning. Reasons for exclusion because of surgical delay included late or missed diagnosis, secondary displacement after the first follow-up visit, patients’ issues (i.e. insurance problems, no show) or system defaults (incorrect merge of diagnosis-code with operation); and (3) caused a presentation delay (delay between injury and first hospital visit) of 14 days or more, as these patients could never be operated on within 14 days after injury.

Before the implementation of the VFC workflow, patients either presented themselves at the ED or scheduled a first appointment at the outpatient clinic. In the VFC period, patients could also visit the plaster room after receiving radiological confirmation of a fracture after referral from a general practitioner. Patients were included in the VFC workflow if they presented through the ED or through the plaster room, had the ability to comprehend either the Dutch or English language, and had a functional phone number. Patients who presented at the outpatient clinic were not subjected to the VFC workflow after VFC implementation. Many had been injured while on holiday, received casting or original treatment at the holiday destination and were referred for further medical attention in their home country. These patients experience a longer interval between their injury and hospital presentation than patients presenting at the emergency department Geerdink et al. (2021b) published a detailed description of the pre-VFC and during-VFC workflow implemented in the study centre. All patients included in the VFC workflow were automatically discussed on the morning of the following weekday in a VFC review meeting, where a multidisciplinary team (i.e. orthopaedic surgeon, surgical residents, plaster room technician, outpatient clinic secretary and surgery scheduler) assigned pre-defined specific treatment plans to each patient, which were then adjusted to fit each patient's specific needs. The pre-defined VFC-review treatment plans were based on national fracture care guidelines and joint consensus of the orthopaedic trauma staff. For surgical treatment specifically, the VFC-review treatment pathways included information on the surgical procedure, the complete follow-up treatment and the recovery process. The VFC team could adjust treatment protocols for each patient based on expert opinion and individual patient’s characteristics (e.g. age, pre-existing function, timing of injury and preferred time of surgery or follow-up).

Direct supervision of this review was under the responsibility of a team of experienced orthopaedic trauma surgeons, including surgeons specialized in hand and wrist care (Geerdink et al., 2021b). All patients discussed in the review were contacted directly afterward by one of the attendees under supervision of the orthopaedic surgeon for explanation of their treatment plan and information regarding their scheduled appointments.

### Data collection

Study data were extracted from an existing database, which contained previously collected data from electronic patient records as part of a quality evaluation audit of the VFC. All study data were stored on a separate, secured hospital drive. Collected data included age, sex, date and time of hospital presentation, date and time of surgical intervention, date of injury, injury type, type of surgery (closed reduction [CR] or open reduction internal fixation [ORIF]), number of hospital contacts, radiographic imaging (radiograph, magnetic resonance imaging [MRI], computed tomography [CT], ultrasound), fracture reductions, number of cast immobilizations and recorded complications.

### Data analysis

The primary outcome was the time between injury and surgery. To determine the exact time to surgery, the difference in days between date and time of first hospital presentation and date and time of the surgical intervention was calculated at a continuous scale. Subsequently, the number of days between the injury and the first hospital presentation was gathered from the patient’s electronic medical history. These days of presentation delay were subsequently added to the previously determined exact days between the first hospital presentation and surgery. In addition, an extra 12 hours were allocated to each patient to fully account for the uncertainty of the exact time of injury.

Secondary outcomes included the quality of the planning of semi-acute surgery. Operations performed within 1–7 days after injury were considered as planned optimally, within 8–10 days were acceptable, 11–14 days as borderline acceptable and more than 14 days as unacceptable.

Primary and secondary outcomes were also assessed separately for children and adolescents aged under 18 years and adults. In addition, the primary outcome was assessed distinctively for CR and ORIF surgeries.

Tertiary outcomes were the number of hospital visits, number of diagnostic tests (radiographs, MRI, CT and ultrasound), number of fracture repositions and number of cast immobilizations. Registered complications were noted and further discussed.

### Statistical analysis

Differences in baseline characteristics between the pre-VFC and VFC cohorts were compared with independent sample *t*-tests or Mann–Whitney U-tests for continuous data, depending on data distributions. Normal distribution of data was established by visual inspection and the Shapiro–Wilk test. In case of normally distributed data, the mean and standard deviation were reported. In case of non-normally distributed data, the median and interquartile ranges (IQR) were reported. chi-squared test were used for categorical data, and Fisher’s exact tests in case of zero cell counts.

The difference between the pre-VFC and VFC cohorts for days until semi-acute surgery as the primary outcome was assessed using the distribution-free log-rank test following the Kaplan–Meier survival analysis. These data were plotted as the increasing proportion of already operated patients over consecutive days since injury in each study group or the cumulative probabilities. With each patient eventually having received an operation, the mean days until semi-acute surgery with its two-sided 95% confidence interval (95% CI) was reported for each group. Cox regression analyses were used to estimate the hazard ratio (HR) of semi-acute surgery in the whole group as well as stratified by adulthood (with 18 years of age as cut-off), thereby quantifying the impact of VFC implementation. The HR reflects the probability for a patient of being operated at any point in time during the VFC period relative to that probability for a patient during the pre-VFC-period. A HR of 1 means that patients in the VFC and pre-VFC periods have a similar probability of being operated at any day. The higher the HR, the sooner patients were operated during the VFC period. The HR is assumed to be constant over time. This proportional hazards assumption was checked by non-crossing of log-minus-log curves and non-significant time-by-covariate interaction in time-dependent Cox regression analysis.

The Cox regression analysis for adults was repeated only for patients who presented at the ED. Patients who presented at the plaster room during the VFC period upon referral by the general practitioner were excluded from this analysis as no such access route was available in the pre-VFC period. They too experienced an increased presentation delay. Patients who presented at the outpatient clinic were not subjected to the VFC workflow.

The linear-by-linear association test for ordered categories was used to detect differences in quality of timely planning operations between pre-VFC and VFC cohorts.

A *p*-value <0.05 indicated statistical significance of differences in the primary and secondary outcomes. Tertiary outcomes were reported descriptively.

## Results

### Patients and baseline characteristics

**Figure 1.**
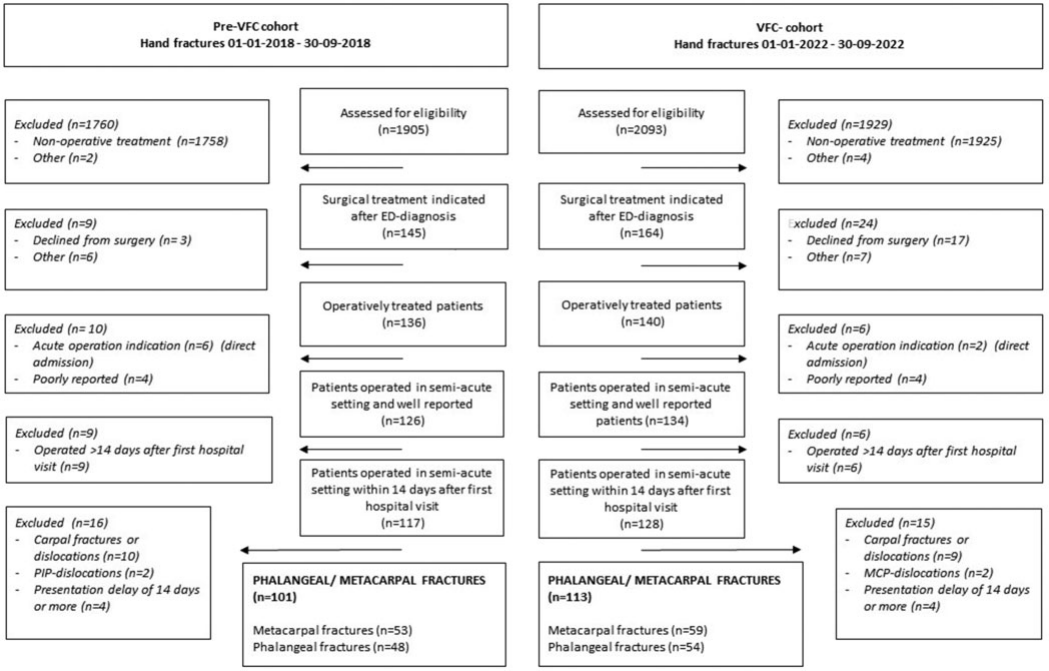
Flow diagram.

Figure 1 shows the flow diagram of patients included in the pre-VFC and VFC cohorts. Overall, 93 (82%) of 113 patients were included in the VFC workflow. The median age of all 214 included patients with phalangeal or metacarpal fractures was 29 years (IQR 22–41), 27% were women and 71% were treated with CR. The median number of days of presentation delay was 0.5 days (IQR 0.5–2.5). There were no significant differences in baseline characteristics between the pre-VFC and VFC cohorts (Table 1).

**Table 1.** Baseline characteristics.

	Phalangeal and metacarpal fractures		*p*-value
	2018 (*n* = 101)	2022 (*n* = 113)	
Age (years)	30 (22–42)	28 (22–41)	0.61
No. of women	32 (32)	26 (23)	0.15
No. of CR	68 (67)	84 (74)	0.26
Presentation delay (days)	0.5 (0.5–2.5)	0.5 (0.5–2.5)	0.60
Optimally planned (1–7 days)	0.5 (0.5–1.5)	0.5 (0.5–1.5)	0.91
Acceptably planned (8–10 days)	0.5 (0.5–1.5)	0.5 (0.5–4.5)	0.10
Borderline acceptable (11–14 days)	1.5 (0.5–4.5)	4.0 (0.5–7.5)	0.33
Unacceptably planned (>14 days)	3.5 (1.5–7.5)	8.5 (6.8–9.8)	0.07
No. of patients per hospital type entrance			
ED	93 (92)	87 (77)	
VFC workflow	Not applicable	79 (91)	
Outpatient clinic	8 (8)	12 (11)	
VFC workflow	Not applicable	0 (0)	
Plaster room	Not applicable	14 (12)	
VFC workflow	Not applicable	14 (100)	

Data are expressed as n (%) or median (IQR).

CR: closed reduction; ED: emergency department; IQR: interquartile range; VFC: virtual fracture clinic.

### Primary and secondary outcomes

**Figure 2.**
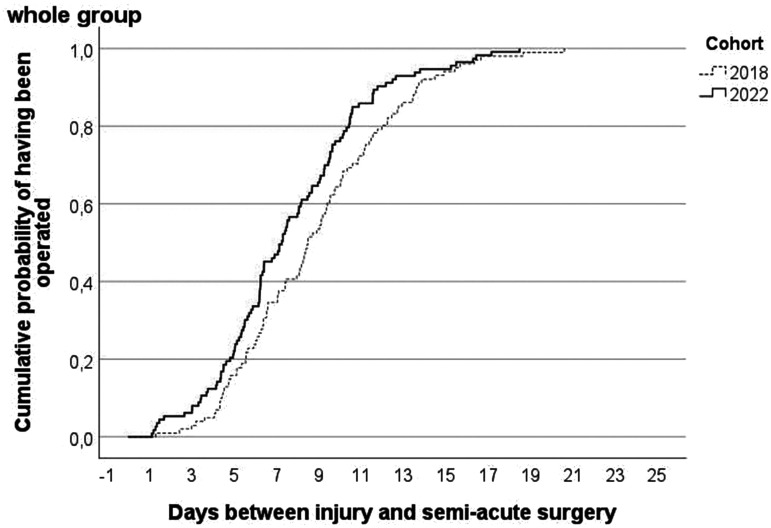
Cumulative probability by virtual fracture clinic-cohort of having received semi-acute surgery after day of injury with phalangeal or metacarpal fracture.

Figure 2 shows the cumulative probability of having received semi-acute surgery by day since injury. Before VFC implementation, patients were operated after 8.9 days, while during the VFC period it took a mean of 7.6 days (*p* = 0.03).

**Figure 3.**
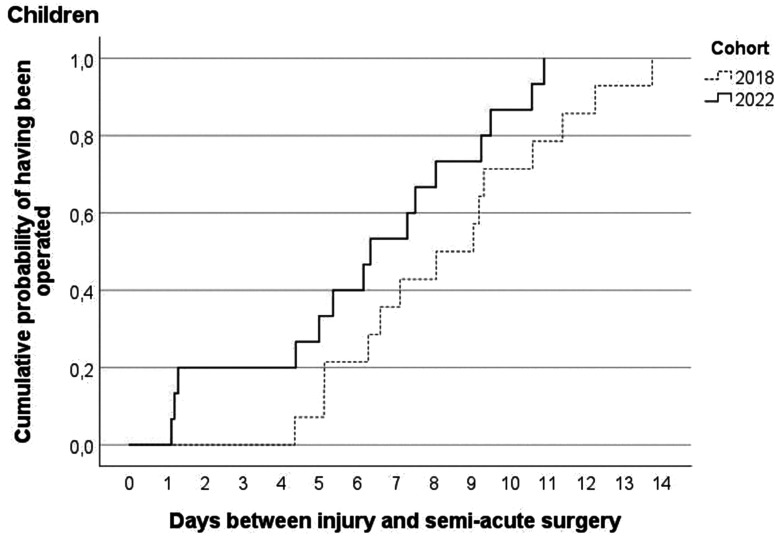
Cumulative probability for children by virtual fracture clinic-cohort of having received semi-acute surgery after day of injury with phalangeal or metacarpal fracture.

**Figure 4.**
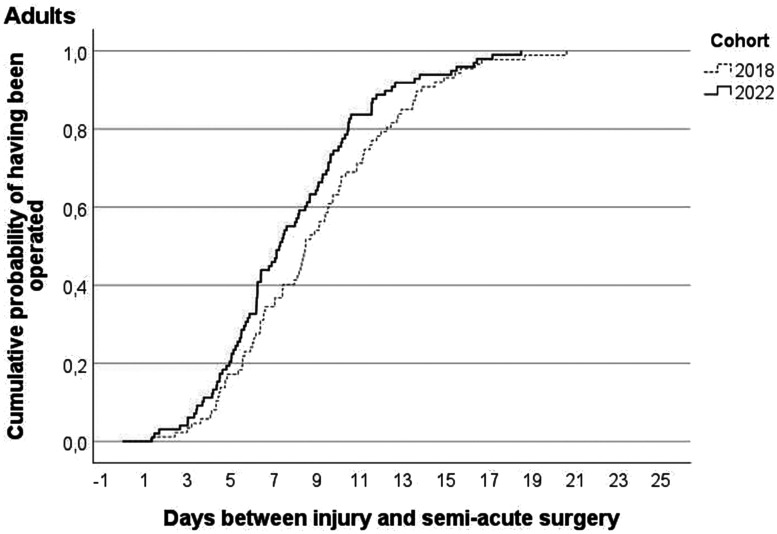
Cumulative probability for adults by virtual fracture clinic-cohort of having received semi-acute surgery after day of injury with phalangeal or metacarpal fracture.

Figures 3 and 4 show the cumulative probabilities of having received semi-acute surgery by day since presentation for children and adults, respectively. On average, children were operated after 8.4 days before and 6.3 days during the VFC period (*p* = 0.09), while adults were operated after a mean of 8.9 days before and a mean of 7.9 days during the VFC period (*p* = 0.06). After excluding those adults who presented at the outpatient clinic or the plaster room, the adults were operated after a mean of 8.7 days before and a mean of 7.4 days during the VFC period (*p* = 0.003).

Patients treated with CR were operated after 8.0 days on average and patients treated with ORIF after 8.7 days (*p* = 0.28). Patients treated with CR were operated after 9.0 days before and 7.2 days during the VFC period (*p* = 0.01). Patients treated with ORIF were operated after a mean of 8.6 days before and a mean of 8.8 days during the VFC period (*p* = 0.97).

More details of the primary and secondary outcomes (number of the subgroups, 95% CI and hazard ratio) are presented in Table S1.

In 2018, 41% (41/101) of the patients were operated within 7 days and 69% (70/101) within 10 days after injury, while in 2022, 54% (61/113) were operated within 7 days and 82% (93/113) within 10 days. The delay was borderline acceptable for 24% (24/101) in 2018 and 12% (14/113) in 2022; hence, 7% (7/101) of the patients was unacceptably planned in 2018, this was 5% (6/113) in 2022 (*p* = 0.03).

### Tertiary outcomes

The median number of hospital contacts per patient, including ED visits, plaster room visits or follow-up by telephone or video or in person through the outpatient clinic, was 5 (IQR 4–6) before VFC implementation and 4 (IQR 3–5) during the VFC period, while the median number of radiographs per patient dropped from 4 (IQR 3–5) to 3 (IQR 3–4). MRI examinations were carried out in 2 (2%) of 101 patients before VFC implementation and in 1 (1%) of 113 patients in the VFC period, CT scans in 21 (21%) and 28 (25%) patients, and ultrasound in 6 (6%) and 4 (4%) patients, respectively. The median numbers of casts remained stable at 3 (IQR 2–4) before and during the VFC period.

Before VFC implementation, one patient with a wound infection was observed and treated with oral antibiotics for 1 week after K-wire fixation for a fracture of the thumb. The patient recovered well. Three patients in the VFC period experienced a complication: two wound infections and one osteosynthesis failure. The first patient developed cellulitis after K-wire removal for proximal phalangeal fracture of the index finger, requiring intravenous antibiotics and two readmissions and followed by full recovery. The second patient developed an infection 9 days after open reduction and internal plate fixation for an oblique metacarpal fracture of the little finger. Re-operation for inspection and cleaning was performed. This patient received antibiotics intravenously and recovered well. The patient who experienced failure of osteosynthesis after screw-fixation of a metacarpal fracture underwent a second surgery for screw removal and plate fixation and recovered without permanent damage.

## Discussion

This retrospective before-and-after cohort study showed that implementation of a VFC was associated with a mean decrease of 1.3 days between injury and operation and a more than 30% increase in the instantaneous probability for patients with a phalangeal or metacarpal fracture of being operated semi-acutely during the first 2 weeks after injury. The reduced time between injury and operation was primarily accounted for by CR types of surgery. Generally, CR surgery is less complex, takes less time to perform and is therefore easier scheduled than ORIF surgery.

In the present study, significant reductions in time until semi-acute surgery were observed, despite presentation delays after injury by patients up to 10 days. As a result, more than half of all the operations became planned optimally and borderline acceptable planning was reduced to one in ten. In 95 out of 100 patients, surgical intervention was planned within a 14-day timeframe. Qualifying 1–7, 8–10 and 11–14 days after hospital presentation as optimal, acceptable and borderline acceptable, respectively, as time frames for the planning of semi-acute surgery is based on daily practice and therefore subject to debate. We could not find studies assessing functional outcomes per day of delay until operation after hand injury, and publications do not provide a conclusive benchmark for the acceptable time until operation in hand fractures or injuries. The Dutch Guidelines for hand fractures do not explicitly recommend a specific timeframe for the operation. However, they do emphasize the importance of reassessing a potentially unstable injury within 1 week after injury if operative treatment is possibly required (Christodoulou et al., 2023; Dutch Federation of Medical Specialists, 2018). Furthermore, the British Society for Surgery of the Hand states that operation of closed fractures should take place within 7 days or, in case of primary non-operative treatment, within 3 days after a secondary displacement (Christodoulou et al., 2023).

Following these current European guidelines, operating within 10 days seems to be considered as clinically relevant. Therefore, it is the authors’ suggestion, based on the guidelines, that operating within 7 days could be considered as most optimal planning. Due to the implementation of VFC, we were able to accomplish successful surgery within the 10-day acceptable timeframe in 82 out of 100 patients and 95 out of 100 patients within the borderline acceptable timeframe of 14 days. This is possibly an underestimation, attributed to the additional 12 hours allocated per patient to account for uncertainty regarding the time of injury on the day of the injury.

The observed tertiary outcomes suggest a more lenient processing of patients with fewer hospital contacts, radiographs and braces. These findings support previous (international) research on VFCs, which indicated positive outcomes in terms of planning and treatment effectiveness in all sorts of trauma-related extremity injuries (Bhattacharyya et al., 2017; Cavka et al., 2021; Davey et al., 2020; Geerdink et al., 2021a, 2021b; McKirdy and Imbuldeniya, 2017; O’Driscoll et al., 2022; Rhind et al., 2020).

The effectivity of a VFC on the patients in this study seems to be threefold. First, the daily VFC review provides the opportunity of early decision making for operative treatment since the review is supervised by a specialized surgeon authorized for decision making upon surgical intervention. The percentage of patients operated with a borderline acceptable delay decreased from 24% to 12%. Second, the planning of surgical treatment and follow-up appointments was more structured and more in advance, leaving room for optimization of schedules and reducing unnecessary appointments. Third, the VFC appears to have the capability to effectively manage patient-presentation delays. The median number of days in presentation delay for patients within the borderline acceptable timeframe was 1.5 days in 2018 compared to 4.0 days in 2022. Meanwhile, for those in the unacceptable timeframe, the respective figures were 3.5 days and 8.5 days. Complications were rare and the impact on health was temporary.

The strength of this study lies in its assessment of the VFC implementation group, mirroring the pre-VFC group ‘as it is’. After the implementation and a reasonable adjustment period to this new workflow, we evaluated all consecutive patients who presented to the hospital and underwent semi-acute operative treatment for hand injuries. The results of this study were obtained with an 80% compliance rate of the VFC workflow during the post-VFC period. It is hypothesized that subgroup analysis focusing solely on VFC-workflow patients may yield even more promising results. However, achieving a scenario where the VFC workflow can be applied to all patients is not currently feasible. The results of this study can be translated realistically into clinical practice.

Another strength of this study is that the time until surgery is determined by the actual duration between the injury and surgery, rather than the time between hospital presentation and surgery. Using the time until surgery from the first hospital presentation would offer a more accurate depiction of the capabilities of a VFC. However, even though presentation delay is beyond the hospital’s control, hospitals and planning must address this real-life scenario.

One limitation of this study is the small sample size of children. Implementation of VFC was associated with a mean decrease of 2.1 days between injury and surgery. It fell short of statistical significance. Another limitation is its retrospective design with the inherent risk of bias. Another limitation of this retrospective before-and-after study concerns the lack of an external control group to distinguish between background trends or external developments over calendar years (e.g. dealing with scarcity during the COVID pandemic) apart from the implementation of a VFC approach. Such a control group could consist of 2018 and 2022 data from other hospitals that have not yet introduced and implemented the VFC concept. A similar combination of a before-and-after design and external control group was successfully applied while assessing the effects of a comprehensive surgical safety system on patient outcomes (de Vries et al., 2010). Notwithstanding the lack of a control group, the suggestion that VFC implementation may have contributed substantially to the reported reduction in time until semi-acute surgery in the present paper is substantiated with reports by others. For example, a shortening of time from electronic VFC assessment to operative treatment was reported from 13 to 9 days in a large UK district general hospital (Sephton et al., 2022). Even though this UK study was not fully comparable to ours because it addressed all types of trauma, the beneficial potential of VFC implementation on preventing undue delay of semi-acute surgery clearly emerges from both studies. These data can be of value for further development and dissemination of VFCs internationally.

## Supplemental Material

sj-pdf-1-jhs-10.1177_17531934241268976 - Supplemental material for The impact on time between injury and semi-acute surgery for hand fractures after virtual fracture clinic implementation Supplemental material, sj-pdf-1-jhs-10.1177_17531934241268976 for The impact on time between injury and semi-acute surgery for hand fractures after virtual fracture clinic implementation by Dorien A. Salentijn, Gijs J.A. Willinge, Marcel G.W. Dijkgraaf and Ruben N. van Veen in Journal of Hand Surgery (European Volume)
