# Supplementary material for: The impact on time between injury and semi-acute surgery for hand fractures after virtual fracture clinic implementation
Source: J Hand Surg Eur Vol. 2024 Aug 21;50(2):169–77. doi: 10.1177/17531934241268976 (PMC11827275; doi:10.1177/17531934241268976)
Supplement: sj-pdf-1-jhs-10.1177_17531934241268976 - Supplemental material for The impact on time between injury and semi-acute surgery for hand fractures after virtual fracture clinic implementation [file sj-pdf-1-jhs-10.1177_17531934241268976.pdf]

Table S1. Time between injury and semi-acute surgery by treatment group and corresponding hazard ratio's

	2018	2022	*p*-value	Hazard Ratio	*p*-value
	Days **(**95% CI)				
Whole group	8.9 (8.1-9.6; N=101)	7.6 (7.0-8.3; N=113)	0.03	1.35 (1.03-1.77)	0.03
Children	8.4 (6.9-9.9; N=14)	6.3 (4.6-7.9; N=15)	0.09	1.94 (0.88-4.26)	0.1
Adults	8.9 (8.1-9.8; N=87)	7.9 (7.2-8.6; N=98)	0.06	1.32 (0.98-1.76)	0.07
ED-visit only	8.7 (7.9-9.6; N=81)	7.4 (6.6-8.1; N=74)	0.003	1.43 (1.04-1.96)	0.03
CR	9.0 (8.1-9.9; N=68)	7.2 (6.5-8.0; N=84)	0.01	1.54 (1.12-2.13)	0.009
ORIF	8.6 (7.3-10.0; N=33)	8.8 (7.5-10.1; N=29)	0.97	0.99 (0.60-1.64)	0.97

ED: emergency department, CR: closed reduction, ORIF: open reduction internal fixation
